# A Study Protocol to Assess the Respiratory Health Risks and Impacts amongst Informal Street Food Vendors in the Inner City of Johannesburg, South Africa

**DOI:** 10.3390/ijerph182111320

**Published:** 2021-10-28

**Authors:** Maasago Mercy Sepadi, Vusumuzi Nkosi

**Affiliations:** 1 Department of Environmental Health, Faculty of Health Sciences, University of Johannesburg, Cnr Sherwell and Beit Street, John Orr Building, 7th Floor, Doornfontein Campus, Doornfontein, Johannesburg 2094, South Africa; vusi.nkosi@mrc.ac.za; 2 Environment and Health Research Unit, South African Medical Research Council, Johannesburg 2094, South Africa; 3 School of Health Systems and Public Health, Faculty of Health Sciences, University of Pretoria, Pretoria 001, South Africa

**Keywords:** South Africa, Johannesburg, respiratory symptoms and diseases, risk assessment, informal, street vendors, occupational health, environmental health

## Abstract

The overall unemployment rate in South Africa was impacted by the coronavirus (COVID-19) pandemic, which led many people to resort to informal work such as street trading opportunities in big cities. However, this work is located in the same cities where air pollution is of concern. Furthermore, literature has indicated the lack of regulation of the informal trading sector as compared to the formal sector. An analytical cross-sectional study is proposed to be conducted amongst all of the informal food street vendors (indoor/inside buildings and outdoor/street pavements stalls) in the inner city of Johannesburg, South Africa. By adopting a total sampling method of 746 vendor stalls, this study’s key focus is on inhalation as an occupational exposure. In addition, the study aims to assess the respiratory risk factors amongst informal food street vendors’ stalls and their impact on vendors’ respiratory health. The risk factors to be assessed include the five common air pollutants: street vendor’s infrastructure; socioeconomic factors; personal behavior such as tobacco smoking and handwashing practices; wearing of respiratory protective equipment; and vendors’ exposure duration. The data collection will follow three phases using quantitative methods. In the pre-assessment phase, it will include a pilot study to test the walkthrough survey checklist and the respiratory symptoms and diseases questionnaire. The assessment phase includes a total of eight area samples, which will be taken in a 1-day event over four yearly seasons, as well as thirty personal samples taken in winter over an 8-h work shift. The post-assessment phase will be the development of a risk impact assessment and a risk management model. The study is essential for healthy occupational conditions as indicated in the Occupational Health and Safety (OHS) Act (no. 85 of 1993) and the Regulations governing general hygiene requirements for food premises, the transport of food, and related matters (no. R638 of 22 June 2018).

## 1. Introduction

In sub-Saharan Africa, including Southern Africa, it was stated that 89% of employment is informal [1]. Many people resort to informal sector employment for various reasons such as lack of jobs or poor educational background, with informal trading as one of these many jobs [2]. In the first quarter of 2021, South Africa’s (SA) Labor Force Survey reported an unemployment rate of 32.6% (about 7.2 million unemployed persons) [3]. The youth aged 15 to 24 years and 25 to 34 years recorded the highest unemployment rate of 63.3% and 41.3%, respectively, with 35 to 44 years at 27%, 45 to 54 years at 20%, and the lowest were 55 to 64 years of age at 13.1% [3]. A comparison of employment in the provinces of South Africa (SA) showed that the highest decline in employment was in the Limpopo province (at 10.0%), followed by Gauteng with a decline of 9.9%. The percentages of persons with educational levels below matric were 52.4%, followed by those with matric at 37.7%, and only 2.1% of unemployed persons were graduates, while 7.5% had other tertiary qualifications as their highest level of education [3]. According to the second quarter statistics, an employment decrease was observed only in the formal sector (375,000), while employment gains were observed in the informal sector (184,000), agricultural sector (69,000), and private households (67,000) [3]. 

The term “informal vendors” refers to the self-employed workers who occupy sidewalks or informal structures. The informal trading by-laws of the City of Johannesburg (COJ) [4] define an informal vendor as “a person who sells goods or services in a designated area”. Furthermore, lifestyle and socioeconomic changes have caused many consumers to find it easier and economical to buy prepared street food [5]. As informal work such as street trading rises in the inner city of Johannesburg, the concern for the health of workers rises, as well. Informal street vendors involved in the preparation and sale of food are the most impacted by the national health legislations [2].

### 1.1. Environmental and Occupational Respiratory Risk Factors of Informal Street Vendors

The respiratory health impact on informal street vendors is due to various occupational risk factors, which include indoor and outdoor air pollutants and other hazards caused by their work environment, operational methods, and personal behavior. An air pollutant is any substance in the air that is harmful to health [6]. The five common air pollutants of concern as per the World Health Organization (WHO) and Centers for Disease Control and Prevention (CDC) are Particulate Matter (PM), Ozone (O_3_), Nitrogen Oxide (NO_2_), Sulfur Dioxide (SO_2_), and Carbon Monoxide (CO) [6,7]. The air quality in urban or major cities all over the world has been problematic, with major air pollution sources such as vehicle emissions and the presence of industrial activities. A street vendor’s study conducted in Bangkok found higher concentrations of PM_2.5_, SO_2_, NO_2_, and CO in roadside work areas as compared to residential work areas [8]. Another study in Hong Kong found a higher concentration of PM_10_ (210 +/− 70 micrograms/m^3^) for outdoor (roadside) vendors when compared to indoor (inside the shops) vendors, which was at (130 +/− 40 micrograms/m^3^) [9]. These results are similar to the findings in Malaysia, where the PM_2.5_ and CO were higher amongst roadside vendors as compared to indoor restaurant workers [10]. 

However, the informal street vendors are also exposed to risk factors from the work environment operations, which contribute to their respiratory health impact. The risk factors include lack of infrastructure (such as a proper shelter to offer protection against environmental nuisances, type of cooking stoves, access to water and hand-wash facilities, improper waste management), exposure to dust, cooking smoke, vector breeding and animal droppings, and general hygiene of the trading stalls and surrounding area. Furthermore, the respiratory health effects can be impacted by the exposure duration, previous work exposures, and previous chronic respiratory diseases. Latency working periods have been studied by various researchers and were proven to have an impact on health. For instance, workers exposed to air pollution may develop chronic respiratory diseases that may occur immediately or only after a few of years [11]. Various traffic related air pollution studies revealed that exposed workers suffer from respiratory morbidity [8,9,10,11,12,13,14,15,16,17] and the risk increases with the higher total exposure period [12].

Other possible risk factors of informal street vendors include socioeconomic factors such as gender, age, education level, and the type of occupation or activity they are involved in. Moreover, personal behavior risk factors include tobacco smoking (active and passive smoking), hand hygiene practices, and wearing of personal protective equipment. 

Good hand hygiene is a key issue when addressing respiratory health and is associated with lowered respiratory infection [18,19,20,21]. However, it is a struggle for street vendors to practice hand hygiene due to the lack of access to water and handwashing facilities. The struggle to adhere to the wearing of Personal Protective Equipment (PPE) is a concern in many workplaces. Respiratory Protective Equipment (RPE) is a type of PPE, which is mainly used to protect workers against inhalation of hazardous substances in the workplace air. For informal street vendors, it is still a concern if they are fully equipped with the knowledge of wearing the proper RPE (depending on the type of hazard they are exposed to), and if there is an adequate maintenance, cleaning, and disinfection of the RPE [14,15,22]. 

### 1.2. Respiratory Health Impacts of Informal Street Vendors

Environmental and occupational risk factors may cause acute and chronic respiratory diseases such as increased incidence of acute upper respiratory infections (eye, nose, and throat irritation) that may interfere with normal activity, increased prevalence or incidence of chest tightness, increased prevalence of wheezing in the chest apart from colds or of wheezing most days or nights, as well as increased prevalence or incidence of cough or phlegm production requiring medical attention [11,23]. In addition, chronic effects include increased mortality, increased incidence of cancer, increased frequency of symptomatic asthma attacks, as well as increased incidence of lower respiratory infections, cancer, stroke, heart, and lung disease, which have prematurely killed about 7 million people yearly [11,23,24]. Furthermore, the World Health Organization (WHO) mentioned that occupational chronic respiratory diseases represent a public health problem with economic implications in all of the countries [11]. 

The street vendor’s workplace or occupational environment puts the vendors at an increased health risk and may contribute significantly to the burden of respiratory diseases. It is estimated that 15% of the population attributable risk of asthma and chronic obstructive pulmonary disease arises from work exposure [11]. The long-term exposure to outdoor air pollution and traffic-related air pollution has been found to shorten life expectancy [11]. Furthermore, the long-term exposure to combustion-related fine particulate air pollution has been found to be a risk factor for cardiac, pulmonary, and chronic bronchitis, as well as decreased lung capacity and lung cancer mortality [11,25,26]. A study on the respiratory system impact of female street vendors in Egypt ranked the bronchial asthma as the highest disease, followed by chronic bronchitis [8]. Another study on street vendors in Bangkok revealed lower respiratory infections (50%) as compared to upper respiratory (37%) [14]. Moreover, insufficient waste disposal facilities and hand wash facilities may have an impact on the informal street vendors. A cross-sectional survey of the exposure to household waste was associated with both increased prevalence of respiratory symptoms (such as coughing, breathlessness, phlegm, sore throat, wheezing) and worse lung function testing results (with self-reported diseases such as Pneumonia, Asthma, Bronchitis, etc.) [27]. 

### 1.3. Informal Vendors in the Public Spaces of South Africa: Practices and Legislation Background

The lack of legislation compliance in street trading activities is a burden to law enforcement officers in SA [28]. However, this challenge is similar to other cities such as China’s rise in unlicensed vendors [29]. Bénit-Gbaffou (2016) reasoned that the poor management of street trading is caused by the contradiction of national policies or frameworks, whose mandate is to fight poverty and promote economic development. However, it fails to address specific municipality issues such as management of dense and congested streets [30].

The key to reducing risks to health caused by occupational or environmental settings is to quantify the health risks and to assess their distribution [26]. This proposed study aims to use quantitative methods to assess the respiratory health risks of indoor and outdoor informal food street vendors and their impact on vendors’ respiratory health.

There is a need for environmental and occupational health interventions for informal street vendors. The following objectives were formulated to achieve the aim of the study:

▪ To identify respiratory risk factors in indoor and outdoor informal food street vendors stalls;
▪ To determine the prevalence of respiratory symptoms and diseases amongst informal food street vendors, and;
▪ To develop an informal vendors’ occupational health and safety model to promote and protect the health of informal street vendors in urban public spaces.

### 1.4. The Significance of the Proposed Study

It is well known that exposure to air pollution is linked to respiratory and other health effects. However, this study’s targeted population is one of the neglected areas of research in terms of environmental and occupational health assessments, as well as interventions implementations. In urban areas and big cities, air pollution is heavily impacted by industrial and construction activities, and heavy traffic [12,13,14,15,16,17]. International research has been extensive on the impact of air pollution on street vendors. However, there are limited quantitative studies on the quality of air in these workers’ occupational spaces. Furthermore, most of the studies focused on the impact of air pollution exposure on respiratory health, while neglecting other respiratory risk factors such as personal behavior, operational methods, work exposure duration, etc. [12,13,14]. Previous SA research studies have focused mostly on the economic matters and operational hardships of informal street vendors. In addition, limited studies exist on the health risks of street vendors [25,26]. Moreover, the related studies on food premises have focused on the street vendor’s knowledge of safe food hygiene and safety principles [5,31,32]. Furthermore, a few studies have focused specifically on the street vendor’s air pollution exposure and its impact on respiratory health. The strength of this proposed study is on closing gaps and increasing knowledge from previous studies using a combination of methods to address the health risks [33] of this novel group (e.g., ambient air measurement and personal exposure, informal vendor’s surroundings and operational methods), as well as on providing an insight on the prevalence of both respiratory symptoms and diseases.

## 2. Materials and Methods

### 2.1. Study Design and Sample

**Figure 1:**
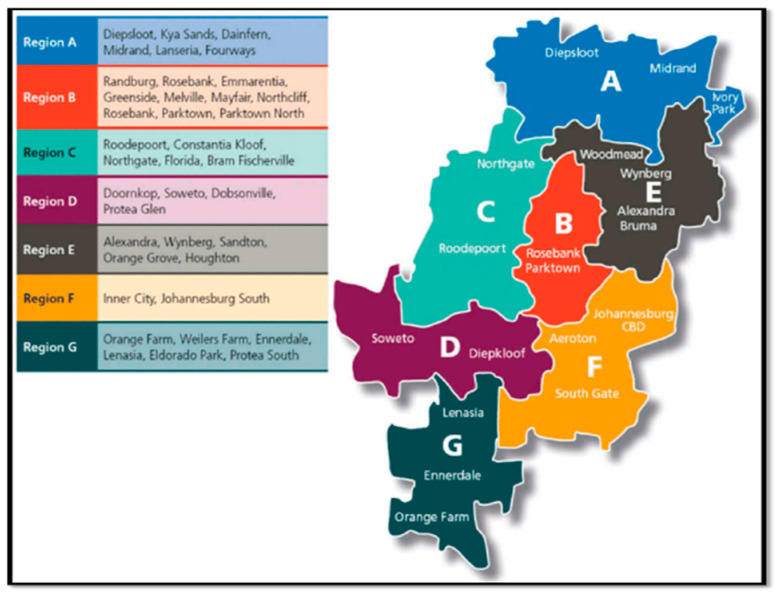
Location map (Region F inner city) (CoJ, 2021).

An analytical cross-sectional study will be conducted at the COJ Metropolitan Municipality (Figure 1), in the Gauteng province of SA. The study will be carried out to compare two types of informal food street vendors population groups, namely indoor (inside a brick and mortar structure) and outdoor (sidewalk pavement), who prepare and sell food. COJ is the largest city in SA and one of the 50 largest urban areas in the world. The city has recorded a population of 5,783,000 in 2020, which is a 2.63% increase from 2019 [34]. The inner city of Johannesburg has a mixture of residential, business, and industrial zoning districts. The 2012 regional distribution of general informal trade activities in Johannesburg indicated that the inner city is a major hub in informal trading [35].

The data collection and analysis will be done using quantitative methods. The limitation of the cross-sectional design that is proposed for this study, is its difficulty to support the causality of potential relationships that may be found. Although the association of the risk factors and ill-health will not be implying direct causal relationships, they will offer some evidence of potential risk factors that could or need to be avoided, in order to prevent the development of respiratory symptoms and diseases amongst informal street vendors.

The strength of this study further lies in the large sample size. A total population sampling will be followed. Involving all of the existing members within the population of interest is one of the advantages of adopting a total population sampling method. This study will give every member a chance to participate in the study and reduce the risk of missing potential insights from members that are not included. Furthermore, this type of sampling makes it possible to make analytical generalizations on the studied population [36]. However, the drawback in this sampling can be the possibility of some street vendors in the target population refusing to participate in the study.

### 2.2. Sample Size Selection

The sampling source for the proposed study is the informal trader database of the COJ Metropolitan Municipality Department of Health (DOH). The sample size for the study will include 746 informal food street vendors (510 outdoor and 236 indoor stalls), who are located in the COJ inner city. However, the researcher will use the updated database at the time of data collection.

#### Inclusion and Exclusion Criteria

The inclusion criterion involves all of the indoor and outdoor informal food street vendors over 18 years, trading in designated daily markets. This includes informal vendors, who prepare their food elsewhere but sell or trade at their stalls.

The exclusion criterion will be all of the informal street vendors outside the inner-city of region COJ and their non-foodstuffs, as well as non-designated street vendors or vendors trading in illegal or prohibited areas, periodic markets, and special events vendors.

### 2.3. Data Collection Instruments

One of the most important steps towards reducing the risk of impaired health, resulting from inhalation of toxic chemicals, is the measurement and evaluation of the hypothesized risk factors and their impact on workers’ respiratory health. The Health Risk and Impact Assessments (HRIA) are programs used by Environmental and Occupational Health and Safety (OHS) professionals to assess the work environment, personal behaviors, social factors, and OHS services [33]. The WHO HRIA process which will be followed during data collection include the identification of hazards using various methods, hazards characterization, exposure assessment, and risk analysis. Furthermore, the following activities will take place during the study.

#### 2.3.1. Pilot Study

A pilot study will be conducted at the Kwa-Mai Mai market to evaluate the suitability of the walkthrough survey checklist (Table S1) and workers’ respiratory health risk factors, symptoms, and diseases interview tools (Table S2). Kwa-Mai Mai is one of the oldest Markets in COJ, located in the Jeppe suburb, between the corner of Anderson and Berea streets [37] with an existing 23 informal food street vendors. The 23 vendors at the market are part of the COJ inner city vendor’s database. The pilot study will be conducted over 3 days between (07:00 to 11:30 a.m.) and (14:00 to 16:00 p.m.) to avoid the informal street vendors rush hour period, which is between (11:30 a.m. to 13:30 p.m.). Each data collection instrument will be used over a single day and an extra day will be given in cases where disruptions occur. The pilot study participants will be excluded from the main study. The walkthrough survey and the interview tools will be adjusted for the main study if challenges or limitations are encountered during the pilot study. This test is necessary to improve the tools where needed before the commencement of a full-scale research project to ensure that the tools suit the study’s target population.

#### Observational Walkthrough Survey Checklist Information

The observational walkthrough survey is an essential step in OHS and environmental health discipline, which is designed to better understand the working conditions and risk factors that workers are exposed to. The food vendor stalls survey checklist questions were derived from the requirements of the OHS Act (no. 85 of 1993) and Regulations (no. R638 of 22 June 2018), as well as from recent similar studies [31,32,38,39]. The data collected during the walkthrough survey will focus on the respiratory risk factors of the trading stalls, which include infrastructure information such as the type of stall (outdoor or indoor) and shelter, work activities performed, cooking equipment; food preparation methods; access to water facilities; general hygiene of the stalls and surrounding area, and waste management; visible vector breeding or animal droppings, e.g., rats, birds; visible dusts; traffic density (light and heavy duty traffic); and the presence of large emission point sources or activities (e.g., power plants, other industrial combustion plants), and the existing control measures in place. The estimated walkthrough time in each vendor’s stall is 10 min. The initial visit arrangement will be made with all of the stall owners or managers.

#### Worker’s Respiratory Health Risk Factors, Symptoms, and Disease Interview

The worldwide validated British Medical Research Council (BMRC) interview will be used as a tool to determine the prevalence of respiratory symptoms and diseases in adults (Table S2). The three-part face-to-face interview will be conducted with close-ended multiple-choice questions. An additional set of questions that identifies each worker’s risk factors that are associated with respiratory health were included using the information from the OHS Act (no. 85 of 1993) and Regulations (no. R638 of 22 June 2018), as well as from similar existing studies [40,41,42].

Section A of the interview will focus on socio-demographic factors such as age, gender, and educational level. Section B will focus on risk factors such as work duration, wearing of PPE, and hand hygiene practices. Since exposure in the home environment may have an impact on health outcomes, indicators such as cooking smoke or fumes at home, living near a heavy trafficked road, living near a large industrial air pollution source, etc., and active and passive smoking were added.

Section C will comprise of upper and lower respiratory symptoms or diseases such as sore throat, eye irritation cough, phlegm, breathlessness, and wheeze and chest illness experienced now and during the past year. The information collected on the diseases refers to respiratory morbidity, for which the respondent received a doctor’s confirmation or diagnosis. It includes chest illnesses such as Bronchitis, Pneumonia, Pleurisy, Pulmonary tuberculosis, Bronchial asthma, and Hay fever resulting in asthma. However, air pollution has been regarded as a pervasive public health issue with cardiac vascular diseases. For a better interpretation of the results, the target illnesses involved cardiac vascular disease (e.g., heart troubles) amongst the other respiratory illnesses mentioned.

Since this study will be conducted during the infamous COVID-19 pandemic, which is a respiratory infection and has symptoms that are similar to other respiratory infections, the participants will be asked if they ever contracted COVID-19. The estimated interview time is 15 to 25 min, with reference to the existing, similar research studies. All of the workers in each stall will be allowed to be part of the interview. The interview will be administered by the researcher and recruited multilingual field assistants in the early morning of the day to avoid disruptions of business during lunch hours. All of the field assistants will be trained for the face-to-face interviews before data collection. The interviews will take place in the vendor’s stalls.

#### 2.3.2. Airborne Pollutants Sampling

The inhalation route of chemical exposure is the most significant concern in the study. Therefore, the airborne sampling program uses two different techniques for supportive evidence. The area sampling will be conducted using the Gillian AirCon-2 stationery or area air sampling pump and the personal sampling will be conducted using the GilAir-3 personal air sampling pump [43,44,45]. The two techniques will be simultaneously collecting the following air pollutants as per WHO guidelines, namely: PM of aerodynamic diameter <10 and 2.5 mm (PM_10_ and PM_2.5_, respectively), O_3_, NO_2_, CO, and SO_2_ [6]. The employed tools and sampling techniques used will comply with the National Institute for Occupational Safety and Health (NIOSH), Occupational Safety and Health Administration (OSHA), and United States Environmental Protection Agency (EPA). The sampling pumps will be set to run over the recommended 8-h work shift. Sampler identification details, calibration details, area sampled, environmental conditions, sampling method, duration of sampling, and weather conditions will be recorded on a recording sheet (Table S3).

#### Environmental or Area Air Sampling (Stationary Sampling)

Area samples are collected to represent the distribution of airborne concentration of a chemical in a specific location or throughout the working area’s general atmosphere. This study focuses on two types of workplaces that are of concern, namely indoor and outdoor informal trading stalls. The stationary samples at the two sites (one indoor stall, Site A and one outdoor stall, Site B) will be carried out during four (1-day) sampling programs in 2021 and 2022, covering particularly the four seasons in SA.

The program will be conducted in spring (between September and November 2021), summer (between December 2021 and February 2022), autumn or fall (between March and May 2022), and winter (between June and August 2022). These separated sampling programs will reflect the influence of seasonally dependent variables [46]. The total samples taken will be eight (four from Site A and four from Site B). These samplers will be placed at stationary or fixed stalls at a recommended height of 1.5 m [43,44,45].

#### Personal or Workers Air Sampling (Activity-Based Sampling)

**Figure 2:**
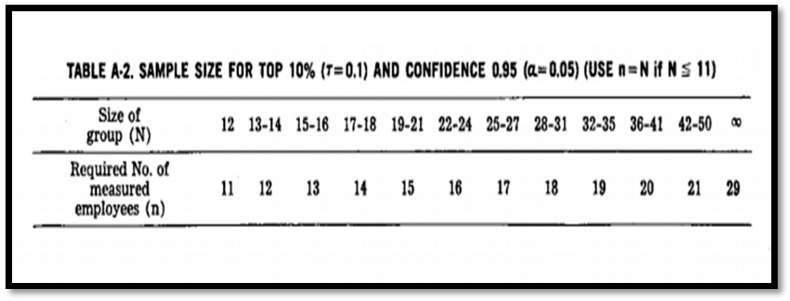
Sample size of the personal air sampling “for a maximum risk subgroup from a homogenous high−risk group” (NIOSH, 1977).

Personal inhalation samples are collected to represent a worker’s inhalation exposure during an entire work shift. In addition, the samples are used to indicate exposure conditions for the rest of the workforce involved in work operations with identical exposure potential. For this study, a random sample size selection will be carried out following the NIOSH occupational exposure sampling strategy manual, which reduces the burden on sampling and analysis resources, while obtaining a high probability of sampling high-risk workers. Figure 2 shows various sample sizes derived from 10% of various population groups, with 95% confidence that at least one individual from the highest 10% exposure group is contained in the sample size [7]. The personal or worker sampling will be conducted amongst 30 informal food vendors (15 from indoor trading stalls and 15 from outdoor trading stalls). This sampling number is above the 29 recommended number of workers measured in a workplace with over 50 employees [7]. However, sufficient information will be obtained after the pre-assessment “walkthrough survey and vendors interview”, which will indicate the type of workers with the highest exposure.

For sampling programs with resource constraints, where the results of a single sampling event will be used for the estimation of chronic exposure, it is recommended that the sampling be conducted under “worst-case” conditions including seasonal considerations [45]. In terms of pollutants exposure in different seasons; most of the industrial emissions remain constant throughout all of the seasons, however the weather favorability differs for each type of pollution [9]. The winter or cold winter season has been found to increase the amount of pollution due to the increase of PM and CO pollutants from activities such as wood burning [9]. Furthermore, air pollution remains on the ground for longer periods in the winter season, resulting in a higher or longer exposure rate as compared to the summer or hot weather [9]. Therefore, the personal sampling for this proposed study will be conducted during the week in the winter season, in order to cover the informal vendors’ busiest operational hours (Monday and Friday) over a single work shift [10,36]. This sampling event will capture the true definition of exposure per participant over the workplace pollutant-generating hours [44].

#### 2.3.3. Post Assessment: Impact Assessment and Management

This stage is crucial to assist in ensuring multi-sectorial (Government, OHS and Environmental Health professionals, and street vendors) responsibility for health and well-being [47]. The HRIA roadmap is a recommended toolkit to estimate the potential impact caused by environmental pollutants on a specified human population system under a specific set of conditions and for a certain timeframe [43,44,45]. The HRIA roadmap will reflect the identified hazards from the airborne sampling and walkthrough survey, known as hazard characterization and exposure assessment, which will reflect the relationship between the dose or amount of pollutant and the probability of developing an illness or health outcome [43,44,45].

The NIOSH risk rating matrix and workers risk profiling methods will be employed. Furthermore, the hierarchy of hazard controls (elimination, substitution, engineering, administrative, PPE) will be followed during the development of health risk mitigations, in response to the informal food street vendors’ exposure assessment.

### 2.4. Data Reliability and Validity

The study will adopt an established BMRC questionnaire with added questions to cover the objectives of this proposed study. Then, a pilot study will be conducted to test the walkthrough checklist and the interview tools to ensure that the questions are appropriate, understandable, and suitable to the target population of informal street vendors [42]. Both personal and area sampling procedures will use reliable and approved methods. A calibration of all the air sampling pumps will be conducted with a spirometer, before and after each use. The information that identifies each sampler will be recorded on a sampling field sheet.

### 2.5. Data Analysis

The walkthrough survey and interview tools are coded before data collection with the assistance of the statistician and supervisor of the University of Johannesburg (UJ) STATKON. All of the data will be exported to the International Business Machines (IBM) SPSS, version 26 software [48]. The walkthrough survey and vendors interview assessment results respond to “Objective I and II” on the identification of other risk factors and respiratory symptoms and diseases. Descriptive statistics will be used to summarize the data, as appropriate. Parametric or non-parametric tests will be applied depending on whether the data is normally distributed or not.

Shapiro Wilk will be applied to test for normality. The prevalence of the health outcomes will be calculated by dividing the number of participants who responded affirmatively to a particular question by the number of interviews completed. Crude and adjusted odds ratios and their 95% confidence intervals will be calculated using univariate and multiple logistic regression to estimate the association between health outcomes and confounding variables. Confounding variables with the *p*-value <0.20 in the univariate regression analyses will be included in the multiple regression analyses. A *p*-value <0.05 in the multiple regression analyses will be considered statistically significant.

The population will be stratified, assuming that the workers in different stalls (indoor/inside buildings and outdoor/street pavements) will exhibit different levels of exposure to air pollutants. Then, the potential airborne contaminants collected in the measurement devices will be quantitatively analyzed in any occupational hygiene approved laboratory. The analysis will be performed using Scanning Electron Microscopy (SEM) that scans a sample with an electron beam to obtain the composition and particle size. The Fourier Transform Infrared (FTIR) spectroscope will be used to calculate the concentration of the elements over eight hours. Moreover, the analysis of the samples will be based on meteorological conditions, e.g., wind, temperature, etc. The air pollution assessment results that respond to “Objective I” will reflect the chemical or air pollutant identification, concentrations, and particle sizes. Once the air pollutants are quantified, a health risk impact and management model will be developed to respond to “Objective II”.

## 3. Study Ethical Considerations

Permission and ethical approval were obtained from the University of Johannesburg (registered as HDC-01-68-2021 and REC-1187-2021), Johannesburg health district, and the City of Johannesburg (registered as NHRD ref no.: GP_202102_036 and DRC ref no.: 2021-02-013). All of the participants will be informed on the aim and methods of the study prior to data collection. Those who agree to participate will be required to give written and signed consent forms. In addition, the interviews will be carried out using the SA indigenous language of choice and participants will be given enough time to respond. The participants may withdraw their consent at any time without any consequences. The identity and confidentiality of all the participants will be protected across all of the activities. This will be done by the use of serial numbers and not the personal details of participants, thus avoiding anyone within or outside of the project from linking the individual’s identity with their responses. Data storage and management will include keeping a hard copy of the records (such as the checklist, interviews, and air sampling forms) in a lockable safe and uploading the records as a soft copy in a cloud storage system, which will be accessible using a password and only available to the University of Johannesburg and the research team.

## 4. Study Dissemination

The findings of the proposed study and their practical significance will be communicated to the participants (informal vendors), the institutions’ management, and other interested stakeholders in an organized meeting. Moreover, the findings will be published and presented at conferences and journals. The thesis will be available on the UJ website.

## 5. Discussion

The United Nations (UN) (2020) recent data from 55 countries showed a median of three deaths and 889 non-fatal injuries occurring per 100,000 employees in the formal sector [49]. However, it is most likely that the informal sector is experiencing a greater risk for the same hazards as compared to their formally employed counterparts [36]. This study sought to highlight the UN (2015) Global Sustainable Development Goals (SDGs), which are more relatable to the objectives of this study [49]. The SDG 3 “ensures healthy lives and promotes well-being” for all. The SDG 3 targets are to reduce one-third of premature mortality by 2030, which is attributed to ambient air pollution and chronic respiratory diseases. The SDG 8 further promotes “decent work and economic growth” and encourages the formalization of small enterprises [49]. Although progress has been slow on these SDGs, it reaffirms the mutually supportive relationship between economic, social, and environmental policies and decent work.

There is a lack of effective legislation that is related to the informal street traders in terms of health and safety and their occupation environment. The regulation governing general hygiene requirements for food premises, the transport of food, and related matters (no. R638 of 22 June 2018) has been developed to regulate food premises [38]. It requires food premises to have a proper infrastructure for the protection of both food handlers and consumers health. The challenge of implementing this same legislation amongst informal vendors places a strain on law enforcement. The same suitable requirements designed for formal businesses should be designed for informal work [36], as they differ in structure and facilities. The COVID-19 OHS guidelines are an example of the requirements that were very challenging to implement in the informal sector as compared to the formal sector.

The OHS legislation should cover workers and employers in all the sectors of the economy and all forms of employment. The OHS Act (no. 85 of 1993) highlights the general duties of employers and self-employed persons in maintaining a safe and risk-free environment for their employees, as well as those who may be directly affected by their work activities [39]. The COJ informal trading by-laws (no. 328 of 2012) stipulate environmental health and safety requirements and violations, which support the existing COJ public health by-laws (no. 179 of 2004) [4,50]. With the number of informal street vendors increasing, the concern for OHS in this sector shows a potential threat to the current efforts of the public health sector.

## 6. Conclusions

This proposed study hopes to identify and increase knowledge on present respiratory health risks and ill-health amongst informal street vendors. The findings of the study will raise awareness of the negative impact of air pollution on street vendors and other occupational hazards that can be detrimental to their respiratory health. The awareness will be achieved through the dissemination of the results. Furthermore, we hope to trigger the thinking points and interaction of occupational health organizations and professionals in developing alternatives and ensuring an improvement in informal trading activities, in order to control risk factors and prevent ill-health amongst this occupational group.

## Data Availability

We did not receive research ethics approval to share raw data publicly. The data that will be collected belongs to the UJ and will be available on reasonable request from the corresponding author.

## Supplementary Materials

The following are available online at https://www.mdpi.com/article/10.3390/ijerph182111320/s1. Table S1: Draft observational walkthrough survey checklist; Table S2: Draft respiratory symptoms and diseases interview; Table S3: Air pollution sampling information sheet.
