# Supplementary material for: A Study Protocol to Assess the Respiratory Health Risks and Impacts amongst Informal Street Food Vendors in the Inner City of Johannesburg, South Africa
Source: Int J Environ Res Public Health. 2021 Oct 28;18(21):11320. doi: 10.3390/ijerph182111320 (PMC8582647; doi:10.3390/ijerph182111320)
Supplement: Supplementary file 1 [file ijerph-18-11320-s001.zip › ijerph-1436098-supplementary.pdf]

Table S1: Draft observational walk-through survey checklist

Stall number and address:	
Inspection date:	

Instruction for the inspector:

- **cycle the correct answer or answers where options are given**

Observation/ matters arising	Items identified	Comments /notes
**GENERAL**		
1. How long has the business existed? Only one answer	1. Less than 2 years<br>2. 2 to years<br>3. to 10 years<br>4. 11 to 15 years<br>5. More than 15 years	
2. How many employees exist in the business? Including owner/manager		
**INFRASTRUCTURE**		
3. Type/location of stall? Only one answer	1. Indoor (inside a building)<br>2. Outdoor (street)	
4. The type of shelter? choose all applicable answers	1.no shelter<br>2.bricks<br>3. corrugated iron sheets<br>4.cardbox<br>5. Other: specify---------------	
5. Do you have access to water?	1.Yes<br>2. No	
6. If yes to Q4. Where are the water facilities?	1.Inside stall<br>2.communal tap<br>3.bring from home<br>4. Other: specify---------------	
**FOOD PREPARATION**		
7. What type of food is sold in your business?	1. Cooked food<br>2. Non-cooked food<br>3. Both	
8. What type of food is sold in your business?, choose all applicable answers	1.Amagwinya/ vetkoek (fried dough bread)<br>2.Bunny chow (hollowed-out /roll bread with e.g. insides of Vienna, sausages, eggs, potato chips)<br>3.Meat<br>4.Porridge/Pap<br>5.Rice<br>6. Soup<br>7.Millie<br>8. Fruits and Vegetables<br>9. Snacks<br>8.Other: specify---------------	
9. Do you prepare (e.g. cooking, packaging) your food onsite (in the stall)?	1. Yes<br>2. No	
9.1. If No, where do you prepare your food?	1. At home<br>2. Another kitchen<br>3. Other specify------	
10. What type of food cooking method/process is done? Choose all applicable options.	1.Grilling<br>2.Boiling<br>3.Frying<br>4.baked<br>5. Other: specify………..<br>7. N/A (non-cooking stalls)	
11. What type of cooking medium fuel do you use? Choose all applicable options.	1. Electric Stove<br>2. Gas stove	

NOTE: This document remains the property of those involved in this study.

	3. Umbawula/wood burning<br>4. Paraffin<br>5. Charcoal<br>6. Other: specify…….<br>7. N/A (non-cooking stalls)	
12. Is there visible smoke during food preparation (exposure to smoke/incense)? Only one option	1. Yes<br>2. No<br>3. N/A (non-cooking stalls)	
**GENERAL HYGIENE OF STALL**		
13. Is the stall food preparation area clean? only one option	1. Yes<br>2. No	
14. Hygiene status of the surrounding area (cleanliness)	1. Yes<br>2. No	
15. Is the stall free from dust? only one option	1. Yes<br>2. No	
16. Is there vector breeding or animal droppings (rats, birds)? Only one option	1. Yes<br>2. No<br>3. N/A (non-cooking stalls)	
17. Is there a waste container with a lid for storage of refuse?	1. Yes<br>2. No	
**OTHER MATTERS**		
18. Traffic density	1. light duty traffic<br>2. heavy duty traffic<br>3. No traffic	
19. Presence of large emission point sources/activities?	1. Yes<br>2. No	
20. If yes to Q19, which sources/activities exist	1. Power plants (electricity generation)<br>2. industrial combustion plants<br>3. construction activities<br>4. Other: please specify………..	
**HEALTH AND SAFETY CONTROLS IN PLACE**		
**Engineering Control**		
21. Any ventilation measure in place?	1. Natural<br>2. Artificial<br>3.Both natural and artificial<br>4. No ventilation<br>5. N/A (no shelter stalls)	
**Administrative controls**		
22. Exposure duration monitored. Choose all applicable options	1. Shift work<br>2. Rotation during preparation<br>3. Other: Specify----	
23. Does the business have any health and safety documents in place? Only one option	1. Yes<br>2. No	____________________
24. If yes to Q11, which type. Choose all applicable options	1. SOP<br>2. H&S policy<br>3. H & S board notice<br>4. Other: specify ……….	____________________
25. Did you ever receive any training on health and safety? Only one option	1. Yes<br>2. No	
26. If yes, on Q13. Whom did you receive training from? Choose all applicable options	1. Department of education<br>2. Department of health<br>3. Department of labour<br>4. In-house training<br>5. Other: Specify…………	
27. If yes to Q13; how frequently do/did you get training? Choose only one option	1. Daily<br>2.. Weekly<br>3. Monthly<br>4. Quarterly<br>5. Twice a year<br>6.Once a year<br>7. Once off	
28. If yes to Q.13; when last did you get the training? Choose only one option	1. Last month<br>2. Last quarter 3. Last year<br>4. Over a year ago	

NOTE: This document remains the property of those involved in this study.

**Air and biological monitoring/examination**		
29. Is air monitoring conducted? Choose only one option	1. Yes<br>2. No	
30. If yes to Q29, which type?	1. Area/ environmental<br>2. Personal<br>3. Both area and personal	
31. If yes to Q.29, by whom? Choose all applicable options	1. Yourself/business<br>2. Private/outsourcing<br>3. Government<br>4. Other stakeholders	
32. If yes to Q.29; when last was it conducted? Choose only one option	1. Last month<br>2. Last quarter<br>3. Last year<br>4. Over a year ago	
33. Is biological examinations conducted for all workers? Choose only one option	1. Yes<br>2. No	
34. If yes to Q.33, whose responsibility is it to conduct the biological examination? Choose all applicable questions	1. By internal worker<br>2. Privately/outsourcing<br>3. As a business	
35. If yes to Q 33, how often are biological examinations conducted within the business? Choose only one option	1. Daily<br>2. Weekly<br>3. Monthly<br>4. Quarterly<br>5. Twice a year<br>6. Once a year<br>7. Once off	
**Personal Protective Equipment's**		
36. Is PPE available?	1. Yes<br>2. No	
37. Does any other health and safety control measures exist?	1. Yes<br>2. No	

NOTE: This document remains the property of those involved in this study.

Table S2: Draft Workers respiratory health risk factors, symptoms and diseases interview

**Interviewer Name(s)**							
**Date of interview**	Day		Month		**Year**		
**Stall type**	Indoor			Outdoor			
**Type of food stall**	Cooked		Non-cooked			Both	
**Stall No/address**							
**Questionnaire serial No.**							
**Other identifying data**							

SECTION A: PARTICIPANTS BIOGRAPHICAL INFORMATION

1. Gender

Male	1	
Female	2	

2. Age

18-24	1	
25-34	2	
35-44	3	
45-54	4	
55-64	5	
65 and older	6	

3. Nationality

South African	1	
Non-South African	2	

4. Educational level

Never attended	1	
Attended primary education (did not complete)	2	
Completed primary education	3	
Attended secondary (did not complete)	4	
Completed secondary education (grade 8-12)	5	
Has post school certificate, diploma or degree	6	

NOTE: This document remains the property of those involved in this study.

SECTION B: OCCUPATIONAL INFORMATION AND RISK FACTORS

Instructions to interviewer

- Put X in appropriate square after each question; When in doubt record "No"
- Write the exact answer of participants in the box on open ended questions.

5. What is your job tittle/ work task? (SELECT ALL APPLICABLE)

Owner/ manager	1	
Chef/cook	2	
Waiter	3	
Other, please specify: ---	4	

6. Approximately how many hours are you working per day?

Less than 8 hours	1	
8 hours	2	
More than 8 hours	3	

7. Are you provided with Respiratory Protective Equipment (RPE)?

Yes	1	
No	2	

8. If yes to Q7, which type of RPE? Tick applicable options

Face shield	1	
N95 mask	2	
Surgical mask	3	
Cloth mask	4	
Other : ---	5	

9. If yes to Q7, how often do you wear or use your RPE?

Never use	1	
Occasionally/sometimes	2	
Almost every time	3	
At all times	4	

10. If yes to Q7, in your work task, how often do you change/wash/disinfect (if cloth mask) your RPE?

Daily	1	
Two to three times per week	2	
Weekly	3	
Every two weeks	4	
Monthly	5	
Quarterly	6	
Annually	7	

11. Do you practice good hand hygiene? Washing hands or sanitizing

Yes	1	
No	2	

12. If yes to Q11, How often do you wash/sanitize your hands while at work?

12.1. Rarely	1	
12.2. Occasionally/sometimes	2	
12.3. Almost every time/often	3	
12.4. At all times/always	4	

13. If yes to Q11, do you wash your hands according to WHO standards? regularly washing hands with soap and water for at least 20 seconds

Yes	1	
No	2	

NOTE: This document remains the property of those involved in this study.

N/A	3	

14. If yes to Q11, do you sanitize your hands according to WHO standards? Using alcohol-based hand sanitizer that contains at least 60% alcohol if soap and water are not available

Yes	1	
No	2	
N/A	3	

15. Other than in this occupation (tick all applicable options)

If you work indoor informal trading: - Have you ever worked in an outdoor informal trading?	1	
If you work outdoor informal trading: - Have you ever worked in an indoor informal trading?	2	
Do you cook at home?	3	

16. Tobacco smoking (tick all applicable options)

16.1.	Do you smoke?	Yes	No
16.2.	If no to 17.1, have you ever smoked?	Yes	No
16.3.	Are you exposed to passive smoking at work or at home?	Yes	No

17. Sources of exposure at the home (tick all applicable options)

17.1.	Cooking smoke/fumes at home	Yes	No
17.2.	Living near a heavy trafficked road	Yes	No
17.3.	Living near large industrial air pollution source	Yes	No

NOTE: This document remains the property of those involved in this study.

SECTION C: RESPIRATORY SYMPTOMS AND DISISEASES

The respiratory questions were adopted from British medical research council and few questions added to suit the objective of the study.

Instructions to interviewer:

- Use actual wording of each question
- Put X in appropriate square after each question
- When in doubt record “No”

Instruction/ information for participants:

- **PREAMBLE:** I am going to ask you some questions, mainly about your chest. I would like you to answer YES, NO OR NOT APPLICABLE.
- All symptoms should have developed since working in this job?

**UPPER RESPIRATORY SYMPTOM/DISEASE**<br>**Note: Since starting this job**				
**1. COUGH**				
1.1.	Do you usually cough first thing in the morning in winter?	**Yes**	**No**	**N/A**
1.2.	Do you usually cough during the day or at night in the winter?	**Yes**	**No**	**N/A**
1.3.	If yes to Q12.1. Or Q12.2: do you cough like this on most days for as much as three months each year?	**Yes**	**No**	**N/A**
**2. PHLEGM**				
2.1.	Do you usually bring up any phlegm from your chest first thing in the morning in the winter?	**Yes**	**No**	**N/A**
2.2.	Do you usually bring up any phlegm from your chest during the day or at night in the winter?	**Yes**	**No**	**N/A**
2.3.	If yes to Q13.1 or Q13.2: do you bring up phlegm like this on most days for as much as three months each year?	**Yes**	**No**	**N/A**
**3. PERIODS OF COUGH AND PHLEGM**				
3.1.	In the past three years, have you had a period of (increased) cough and phlegm lasting for three weeks or more?	**Yes**	**No**	**N/A**
3.2.	If yes to Q14.1, have you had a period of (increased) cough and phlegm lasting for three weeks or more, for more than three winters (since starting this job)?	**Yes**	**No**	**N/A**
**4. BREATHLESSNESS**<br>**Note: if the participant is disabled from walking by any condition other than heart or lung disease, omit Q4.1- 4.3**				
4.1.	Are you troubled by shortness of breath when hurrying on level ground or walking up a slight hill?	**Yes**	**No**	**N/A**
4.2.	If yes to Q4.1: Do you get short of breath when walking with others of your own age on level ground on level ground?	**Yes**	**No**	**N/A**
4.3.	If yes to Q4.1. Do you have to stop for breath when walking at your own pace on level ground?	**Yes**	**No**	**N/A**
**5. WHEEZING**				
5.1.	Have you had attacks of wheezing or whistling in your chest at any time in the last 12 months?	**Yes**	**No**	**N/A**
5.2.	Have you ever had attacks of shortness of breath with wheezing?	**Yes**	**No**	**N/A**
5.3.	If yes to Q16.1: is/was your breathing absolutely normal between attacks?	**Yes**	**No**	**N/A**
5.4.	Have you at any time in the last 12 months been woken at night by an attack of shortness of breath?	**Yes**	**No**	**N/A**
**6. CHEST ILLNESSES**				
6.1.	During the past three years have you had any chest illness which has kept you from your usual activities for as much as a week or longer?	**Yes**	**No**	**N/A**
**6.2.**	If yes to 61:, which chest illnesses (all applicable options) kept you from your usual activities for as much as a week or longer(based on medical diagnosis):			
Heart trouble or Other Vascular Diseases		**1**		
Bronchitis		**2**		
Pneumonia		**3**		
Pleurisy		**4**		
Pulmonary tuberculosis		**5**		
Bronchial asthma		**6**		
Hay fever resulting in asthma		**7**		

Other chest trouble	**8**		
6.3. If yes to Q21.1: Did you bring up phlegm more than usual in any of these illnesses?	**Yes**	**No**	**N/A**
6.4. If yes to Q21.1: Have you had more than 1 illness like this in the past three years?	**Yes**	**No**	**N/A**
**7. PAST ILLNESSES**			
**7.1.** Have you ever been medically assessed/examined or diagnosed with any of the following illnesses (choose applicable options):			
An injury or operation affecting your chest	**1**		
Heart trouble	**2**		
Bronchitis	**3**		
Pneumonia	**4**		
Pleurisy	**5**		
Pulmonary tuberculosis	**6**		
Bronchial asthma	**7**		
Hay fever resulting in asthma	**8**		
Other chest trouble: Specify------------------------	**9**		
**8. LOWER RESPIRATORY SYMPTOMS OTHERS**			
**8.1.** Which of these symptoms do you experience since working in this Job? Cross all the applicable options:			
Nasal congestion	**1**		
Sore throat	**2**		
Cold	**3**		
N/A	**4**		
**8.2.** How often do you have an eye, nose or throat irritation while cooking e.g. any tears or runny nose? Cross the only one applicable option:			
Never	**1**		
Rarely	**2**		
Sometimes	**3**		
Often	**4**		
Always	**5**		
**9. COVID19 related matters**			
24.1. Did you ever get infected with COVID-19 between the onset of the pandemic and now? (only medical confirmed cases)	**Yes**	**No**	

NOTE: This document remains the property of those involved in this study.

Table S3: Air pollution sampling information sheet

The air pollution sampling sheet was adopted from (NIOSH occupational exposure sampling strategy manual, 1977) and few items added to suit the objective of the study.

A. ENVIRONMENTAL CONDITION

Sampling geographical location:								
Date of sampling:								
Season	spring		summer		Autumn		winter	
Temperature:								
Wind Speed:								
Wind Direction:								

B. SAMPLING METHOD/TYPE:

Fixed area	1
Personal/worker	2

C. IF PERSONAL TYPE, WHICH WORK ENVIRONMENT DOES THE WORKER BELONGS TO?

Informal (outdoor) vendor:	1
Informal (indoor)vendor	2

D. **DUST SAMPLER IDENTIFICATION DETAILS:**

Pump No:	
Filter unique No	
Blank sampler:	
Sample work-station/Location/ Area/ identity number of personnel:	
Pump Start Time:	

E. CALIBRATION INFORMATION

Calibration location:	
Calibration date:	
Indicated flow rate:	
Volume :	

F. AIR MONITORING DURATION

Total duration	
Pump Start Time:	
Pump Stop Time:	

G. **SAMPLING/ANALYTICAL METHOD**

Total duration	
Pump Start Time:	
Pump Stop Time:	

H. REMARKS, POSSIBLE INTERFERENCE, ACTION TAKEN, ETC.:

NOTE: This document remains the property of those involved in this study.
